# Calcium-sensing receptor regulates Kv7 channels via G_i/o_ protein signalling and modulates excitability of human induced pluripotent stem cell-derived nociceptive-like neurons

**DOI:** 10.1111/bph.16349

**Published:** 2024-04-16

**Authors:** Nontawat Chuinsiri, Nannapat Siraboriphantakul, Luke Kendall, Polina Yarova, Christopher J. Nile, Bing Song, Ilona Obara, Justin Durham, Vsevolod Telezhkin

**Affiliations:** 1 School of Dental Sciences, Faculty of Medical Sciences, Newcastle University, Newcastle upon Tyne, UK; 2 Translational and Clinical Research Institute, Faculty of Medical Sciences, Newcastle University, Newcastle upon Tyne, UK; 3 Institute of Dentistry, Suranaree University of Technology, Nakhon Ratchasima, Thailand; 4 Oral Health Center, Suranaree University of TechnologyHospital, Suranaree University of Technology, Nakhon Ratchasima, Thailand; 5 Institute of Biomedical and Health Engineering, Shenzhen Institutes of Advanced Technology, Chinese Academy of Sciences, Shenzhen, China; 6 School of Dentistry, College of Biomedical and Life Sciences, Cardiff University, Cardiff, UK; 7 School of Pharmacy, Faculty of Medical Sciences, Newcastle University, Newcastle upon Tyne, UK

**Keywords:** Ca^2+^-sensing receptors, electrophysiology, G protein pathways, Kv7.2/7.3 channels, stem cells

## Abstract

**Background and Purpose:**

Neuropathic pain, a debilitating condition with unmet medical needs, can be characterised as hyperexcitability of nociceptive neurons caused by dysfunction of ion channels. Voltage-gated potassium channels type 7 (Kv7), responsible for maintaining neuronal resting membrane potential and thus excitability, reside under tight control of G protein-coupled receptors (GPCRs). Calcium-sensing receptor (CaSR) is a GPCR that regulates the activity of numerous ion channels, but whether CaSR can control Kv7 channel function has been unexplored until now.

**Experimental Approach:**

Experiments were conducted in recombinant cell models, mouse dorsal root ganglia (DRG) neurons and human induced pluripotent stem cell (hiPSC)-derived nociceptive-like neurons using patch-clamp electrophysiology and molecular biology techniques.

**Key Results:**

Our results demonstrate that CaSR is expressed in recombinant cell models, hiPSC-derived nociceptive-like neurons and mouse DRG neurons, and its activation induced depolarisation via Kv7.2/7.3 channel inhibition. The CaSR-Kv7.2/7.3 channel crosslink was mediated via the G_i/o_ protein-adenylate cyclase-cyclicAMP-protein kinase A signalling cascade. Suppression of CaSR function demonstrated a potential to rescue hiPSC-derived nociceptive-like neurons from algogenic cocktail-induced hyperexcitability.

**Conclusion and Implications:**

This study demonstrates that the CaSR-Kv7.2/7.3 channel crosslink, via a G_i/o_ protein signalling pathway, effectively regulates neuronal excitability, providing a feasible pharmacological target for neuronal hyperexcitability management in neuropathic pain.

## 1 Introduction

Neuropathic pain, defined as a pain caused by a lesion or disease affecting the somatosensory system, exerts significant impacts on individuals' quality of life (Shueb et al., 2015) and causes a serious socio-economic burden (Schaefer et al., 2014). The pathophysiological mechanisms of neuropathic pain involve hyperexcitability of nociceptive neurons associated with dysfunction of neuronal ion channels, such as voltage-gated sodium (Na_V_), voltage-gated potassium (K_V_), and voltage-gated calcium (Ca_V_) channels (Li et al., 2014; Siqueira et al., 2009; Takeda et al., 2011). Carbamazepine, a Na_V_ channel blocker and gabapentinoids, Ca_V_ channel blockers, have been used for neuropathic pain management. However, these medications are not always effective in producing a satisfactory pain reduction (Haviv et al., 2014), suggesting that direct targeting of specific ion channels may not be an optimal solution. Upstream regulatory mechanisms of key ion channels, via G protein-coupled receptors (GPCRs), may be implicated in the pathophysiology of neuronal hyperresponsiveness, suggesting that GPCRs might provide a more appropriate therapeutic avenue for neuropathic pain management (Pan et al., 2008).

Canonical neuronal Kv7 channels (Kv7.2 and Kv7.3 heterotetramers), encoded by the *KCNQ2* and *KCNQ3* genes, specialise in maintaining the resting membrane potential (RMP) and regulating action potential (AP) firing (Peng et al., 2017; Tsantoulas & McMahon, 2014). Dysfunction of Kv7 channels underpins several neuronal hyperexcitability disorders, including epileptic seizures and neuropathic pain (Ling et al., 2017; Nardello et al., 2020; Schroeder et al., 1998). The ionic current passing through Kv7 channels is known as the M-current (*I*_m_), which is mostly conducted through Kv7.2/7.3 channels in neurons (Wang et al., 1998). *I*_m_ is characterised by a continuous voltage-dependent outward current activating at subthreshold firing potentials, approximately −60 mV, and has slow activation-deactivation kinetics (Brown & Adams, 1980; Wang et al., 1998). Several studies have revealed that Kv7 channels can be modulated by GPCRs (Greene & Hoshi, 2017). Muscarinic cholinergic M_1_ receptor activation was first demonstrated to mediate Kv7 channel inhibition via the G_q/11_ protein signalling pathway (Brown & Adams, 1980; Winks et al., 2005). Later, activation of bradykinin B2 receptors and purinergic metabotropic P2Y receptors were shown to induce *I*_m_ inhibition via the G_q/11_ protein (Cruzblanca et al., 1998; Liu et al., 2010; Zaika et al., 2007).

The Ca^2+^-sensing receptor (CaSR) is a GPCR, its main physiological function being the homeostatic maintenance of the extracellular Ca^2+^ concentration ([Ca^2+^]_o_) (Leach et al., 2020). The canonical signalling pathways of CaSR include a G_q/11_ protein-dependent increase in intracellular Ca^2+^ concentration ([Ca^2+^]_i_) (Brown et al., 1993) and a G_i/o_ protein-dependent reduction in 3′,5′-cyclic adenosine monophosphate (cAMP) levels (Chang et al., 1998). Since its original discovery, it has become evident that CaSR is abundantly expressed in the nervous system (Brown & Passmore, 2009; Heyeraas et al., 2008; Ruat et al., 1995), where its dysfunction has been linked to seizures (Kapoor et al., 2008). CaSR can regulate neuronal excitability via several ion channels, for example, non-selective cation channels and Ca^2+^-activated K^+^ channels (Vassilev et al., 1997; Ye et al., 1997). Negative allosteric modulators (NAMs) of CaSR, more commonly known as calcilytics, have demonstrated promising therapeutic uses in certain neurological diseases, for example, traumatic brain injury and Alzheimer's disease (Armato et al., 2013; Xue et al., 2017), whereas positive allosteric modulators (PAMs) or calcimimetics were suggested for neuroblastoma therapy (Rodríguez-Hernández et al., 2016). The role of CaSR in inflammation (Iamartino & Brandi, 2022) also suggests that CaSR might be associated with neuropathic pain, because inflammatory processes are relevant in the pathophysiology of neuropathic pain (Ellis & Bennett, 2013).

Based on the evidence that neuronal hyperexcitability is associated with dysfunctions of CaSR (Kapoor et al., 2008) and neuronal Kv7.2/7.3 channels (Chokvithaya et al., 2023; Schroeder et al., 1998), it is conceivable that a crosslink between CaSR and Kv7.2/7.3 channels exists and contributes to the regulation of neuronal excitability. This study aimed to establish the molecular pathway linking CaSR and Kv7.2/7.3 channels, and to explore the potential of calcilytics in suppressing neuronal hyperexcitability, using a human induced pluripotent stem cell (hiPSC)-derived nociceptive-like neuronal model.

## 2 Methods

### 2.1 Materials

The following compounds and antibodies were used in this study: NPS-R568, a CaSR PAM agonist (Tocris Bioscience, Abingdon, UK); NPS-2143, a CaSR NAM (Tocris); retigabine, a Kv7 channel opener (Tocris); XE991, a Kv7 channel blocker (Tocris); forskolin, a transmembrane adenylate cyclase (AC) activator (Tocris); 8-Br-cAMP, a phosphodiesterase-resistant cAMP analogue and activator of protein kinase A (PKA) (Merck Life Science, Dorset, UK); *Bordetella pertussis* toxin (PTX), a specific inhibitor targeting the alpha subunit of the G_i/o_ protein (Merck); nystatin (Merck); U73122, a phospholipase C (PLC) inhibitor (Tocris); 1,2-Bis(2-aminophenoxy)ethane-N,N,N′,N′-tetraacetic acid tetrakis acetoxymethyl ester (BAPTA-AM), an [Ca^2+^]_i_ chelator (Tocris); NiCl_2_ hexahydrate, a non-selective blocker of Ca^2+^ influx (Fisher Scientific, Loughborough, UK); bradykinin (Merck); substance P (Tocris); adenosine 5'-triphosphate (ATP; Merck); tumour necrosis factor (TNF*α*) (Merck); mouse monoclonal anti-CaSR (Abcam Cat# ab19347, RRID:AB_444867, Abcam, Cambridge, UK); rabbit polyclonal anti-CaSR (Sigma-Aldrich Cat# SAB4503369, RRID: AB_10747268); rabbit polyclonal anti-transient receptor potential vanilloid 1 (Abcam Cat# ab3487, RRID:AB_2209009); rabbit polyclonal anti-*KCNQ2* (Abcam Cat# ab22897, RRID:AB_775890); rabbit polyclonal anti-*KCNQ3* (Thermo Fisher Scientific Cat# PA1–930, RRID:AB_2131708, Life Technologies, Paisley, UK); mouse monoclonal anti-microtubule-associated protein 2 (Sigma-Aldrich Cat# M1406, RRID:AB_477171); rabbit polyclonal anti-peripherin (Abcam Cat# ab4666, RRID:AB_449340); mouse monoclonal anti-phosphoserine conjugated to Duolink^®^ PLA MINUS probe (DUO87012, Merck); Alexa 488 donkey anti-mouse IgG (H + L) highly cross-adsorbed secondary antibody (Thermo Fisher Scientific Cat# A-21202, RRID: AB_141607); Alexa 594 donkey anti-mouse IgG (H + L) highly cross-adsorbed secondary antibody (Thermo Fisher Scientific Cat# A-21203, RRID:AB_2535789); Alexa 488 donkey anti-rabbit IgG (H + L) highly cross-adsorbed secondary antibody (Thermo Fisher Scientific Cat# A-21206, RRID:AB_2535792); Alexa 594 donkey anti-rabbit IgG (H + L) highly cross-adsorbed secondary antibody (Thermo Fisher Scientific Cat# A-21207, RRID:AB_141637); Alexa 488 goat anti-mouse IgG (H + L) pre-adsorbed secondary antibody (Abcam Cat# ab150117, RRID:AB_2688012).

### 2.2 Cell line culture

Chinese hamster ovary cells (RRID: CVCL_0213) stably expressing human Kv7.2/7.3 channels (CHO-Kv7.2/7.3) and human embryonic kidney 293 cells (RRID:CVCL_E339) stably expressing the CaSR (HEK-CaSR) were cultured separately in Gibco™ Dulbecco’s Modified Eagle Medium (DMEM)/Nutrient Mixture F-12 (Life Technologies) supplemented with 10% foetal bovine serum (Life Technologies), 1% L-glutamine (Life Technologies) and 1% penicillin/streptomycin (Life Technologies), and maintained in a humidified incubator at 37°C with 5% CO_2_/95% O_2_. The culture medium was replaced every 2 to 3 days and confluency was assessed with a Leica DM IL inverted microscope (Leitz, Wetzlar, Germany). The cells were passaged using 0.25% trypsin-ethylenediamine tetra acetic acid (EDTA; Life Technologies) upon reaching 80% confluency.

HD33n1 hiPSCs (RRID:CVCL_W557) were cultured in 6-well plates, which were precoated with Corning^®^ Matrigel^®^ basement membrane matrix (Scientific Laboratory Supplies, Nottingham, UK), containing mTeSR™ plus medium (Stemcell technologies, Cambridge, UK) at 37°C with 5% CO_2_/95% O_2_. The culture medium was replaced every 2 to 3 days; the cells were passaged using 0.02% EDTA Ca^2+^/Mg^2+^-free PBS pH 7.2–7.4 (Merck) once 80% confluency was reached. The hiPSCs were differentiated into neurons using a previously published protocol (Telezhkin et al., 2016) with minor modifications: incorporation of 0.33 μL·mL^−1^ ECMAtrix-511 E8 laminin substrate (Merck) into the SCM2 medium and exclusion of LM22A4 from both SCM1 and SCM2 media. After at least 10 days in SCM2 medium, the cells were used for experiments. The SCM1/2-based protocol utilised certain small molecule inhibitors, including SB431542, LDN193189, CHIR-99021 and (2S)-N-[(3,5-Difluorophenyl)acetyl]-L-alanyl-2-phenyl]glycine 1,1-dimethylethylester (DAPT, which were commonly used by several others to generate nociceptive neuron-like cells from hiPSCs (Chambers et al., 2012; Eberhardt et al., 2015; Guimarães et al., 2018). In this study, hiPSC-derived neurons were characterised using patch-clamp electrophysiology (functional assessment) and immunocytochemistry (protein expression of a non-specific neuronal marker MAP 2, a peripheral neuronal marker peripherin, and a predominant marker of human nociceptors, TRPV1) as described below.

### 2.3 Isolation and culture of primary DRG neurons

Animal procedures were approved by the Animal Welfare and Ethical Review Body of Newcastle University. Animal studies are reported in compliance with the ARRIVE guidelines (Percie du Sert et al., 2020) and with the recommendations made by the British Journal of Pharmacology (Lilley et al., 2020). Naïve 6-month-old male CB57BL/6 mice (n = 12 mice, Comparative Biology Centre, Newcastle University, UK) were housed in 500-cm^2^ polycarbonate cages under environmentally controlled conditions (20–24°C, 12-h light–dark cycle, 45%–65% humidities) and with free access to food and water. The mice were killed by cervical dislocation and their dorsal root ganglia (DRG) extracted from all spinal levels under a microscope. The collected DRG were enzymatically digested using collagenase (5 mg·mL^−1^, Merck), papain (5 mg·mL^−1^, Merck), and dispase (10 mg·mL^−1^, Merck) diluted in Ca^2+^/Mg^2+^-free Hanks’ balanced salt solution (Life Technologies) for 40 min (min) at 37°C. The DRG were gently triturated and resuspended in DMEM/Nutrient Mixture F-12. The DRG cell suspension was plated onto circular glass coverslips (Fisher Scientific), which were pre-coated sequentially with poly-D-lysine & laminin (Merck) and Matrigel, and fitted into 24-well plates. The isolated DRG neurons were cultured in vitro in 50% DMEM/Nutrient Mixture F-12 and 50% Neurobasal-A medium (Life Technologies), supplemented with 1% L-glutamine, 1% penicillin/streptomycin, 2% MACS^®^ NeuroBrew-21 (Miltenyi Biotec, Surrey, UK), 2-μM PD0332991 (Tocris), 10-ng·mL^−1^ human brain-derived neurotrophic factor (BDNF) (Miltenyi Biotec), 3-μM CHIR-99021 (Tocris), 200-μM L-ascorbic acid (Merck) and CaCl_2_ (to the final concentration of 1.8 mM). Experiments were carried out three to 14 days after plating.

### 2.4 Immunocytochemistry

Cells (CHO-Kv7.2/7.3, HEK-CaSR, or hiPSC-derived neurons) were plated on 13-mm diameter circular glass coverslips, fixed with 4% paraformaldehyde (Fisher Scientific) for 15 min and washed with phosphate buffer solution (PBS) three times. The fixed cells were blocked and permeabilised with 1% bovine serum albumin (Merck) and 0.1% Triton™ X-100 (Merck) in PBS for 1 h at room temperature. The coverslips were incubated with the primary antibody diluted in the blocking/permeabilisation solution, at a concentration as indicated above, and kept at 4°C overnight. The next day, the coverslips were washed with PBS three times followed by an application of the secondary antibody for 1 h at room temperature. Afterwards, the coverslips were washed with PBS three times before being mounted using ProLong™ Diamond Antifade Mountant with 4′,6-diamidino-2-phenylindole (DAPI; Life Technologies). Images were obtained with Olympus BX61 fluorescence microscope or Zeiss LSM800 laser confocal scanning microscope and processed using cellSens imaging software (Olympus, Tokyo, Japan).

### 2.5 Electrophysiology

Prior to electrophysiological experiments, specialised silicone chambers secured on square glass coverslips (approximate volume of 200 μL) were prepared. CHO-Kv7.2/7.3 or HEK-CaSR were then plated on the prepared chambers and incubated at 37°C with 5% CO_2_/95% O_2_ overnight before conducting any electrophysiological experiments. Circular glass coverslips with mouse DRG neurons cultured in vitro or hiPSC-derived neurons were placed inside the silicone chambers prior to conducting experiments.

The chambers were mounted on a microscope stage and perfused (~500 μL·min^−1^) with standard bath solution containing (in mM): 146 NaCl, 2.5 KCl, 0.5 MgCl_2_, 0.5 CaCl_2_, 10 D-glucose, 5 HEPES; pH was adjusted to 7.4 using NaOH. The cells were patched at room temperature with borosilicate glass pipettes (Harvard Apparatus, Cambourne, UK), with 3 to 7-MΩ resistance when filled, in the conventional whole-cell configuration, except for the recordings of *I*_m_ in mouse DRG neurons when the perforated patch technique using 300-μg·mL^−1^ nystatin was performed. The standard pipette solution contained (in mM): 140 KCl, 6 NaCl, 4 Na_2_-ATP, 3 MgCl_2_, 1 CaCl_2_, 5 HEPES and 5 EGTA; pH was adjusted to 7.2 with KOH. All salts and compounds in the standard bath and pipette solutions were purchased from Fisher Scientific.

The RMP was recorded with the gap free protocol (current = 0 pA) using an Axopatch 200A amplifier and Digidata 1440 A/D interface (Axon Instruments, Forster City, USA). The macroscopic transmembrane *I*_m_ was recorded using a voltage-stepped deactivation protocol, by holding the cells at a potential of −20 mV and stepped down to −60 mV for 4 s. All recordings were filtered at 2 kHz and digitised at 5 kHz. Clampex 10.2 (Molecular Devices, Sunnyvale, USA) was used for data acquisition. Quantification of *I*_m_ was performed during the voltage activation step.

For the determination of the spontaneous activity, cells were held with a current of 0 pA for 1 min to allow for observation of any spontaneous action potentials (sAP) being generated. The cells were categorised into three groups based on the sAP type: quiet, attempting sAP and complete sAP (Figure 6a) (Telezhkin et al., 2016). The ability of the cells to generate induced action potentials (iAPs) was determined using a 1-s current step injection protocol. First, the cells were artificially hyperpolarised and held between −80 and −90 mV. Afterwards, the current step was incrementally increased by 10 pA per sweep up to a maximum of 180 pA; 30 ms was allowed between steps for recovery. Classification of the iAP type was done at the threshold level when the first iAP was produced, and the cells were categorised into three groups: complete single iAP, attempting train of iAP and complete train of iAP (Figure 6c) (Telezhkin et al., 2016). The cells, producing immature iAPs that did not reach 0 mV, were excluded from analyses.

Spike analysis of the first iAP was performed on cells that produced at least a complete single iAP. Using Clampfit 10.2, several characteristics of an iAP were determined as shown in Figure 7a. Some characteristics can be measured directly, including overshoot, afterhyperpolarisation and spike height. To determine the half-height width, the voltage at 50% of the spike height was first measured, and the time difference between which this voltage was reached during depolarisation and repolarisation was calculated. The depolarisation rate and repolarisation rate were defined as the maximum rising slope and maximum declining slope, respectively, which were measured from the first differentiation of voltage with respect to time. The threshold was determined at the peak of the third differentiation of voltage with respect to time during the depolarisation phase of an AP (Henze & Buzsáki, 2001; Telezhkin et al., 2016).

### 2.6 Detection of intracellular cAMP levels and PKA activity

CHO-Kv7.2/7.3 were plated in 12-well plates at a density of 5 × 10^5^ cells per well and incubated at 37°C with 5% CO_2_/95% O_2_. The following day, wells were divided into groups and received appropriate media (Figure 5g,h). The following commercial kits were used to determine intracellular cAMP levels and PKA activity, respectively, according to the manufacturers' instructions: Enzo^®^ Life Sciences Direct cAMP enzyme-linked immunosorbent assay (ELISA) kit (ADI-900-066, Fisher Scientific) and Invitrogen™ PKA Colorimetric Activity kit (EIAPKA, Life Technologies). The intracellular cAMP levels and PKA activity were normalised to total protein concentrations, which were determined using the Pierce™ BCA Protein Assay Kit (Life Technologies) for samples prepared for cAMP detection, and the Pierce™ 660-nm Protein Assay Reagent (Life Technologies) for samples prepared for PKA activity detection according to the manufacturers' instructions. Each assay was performed in duplicate.

### 2.7 Phosphoserine-*KCNQ2* PLA

CHO-Kv7.2/7.3 were plated on 13-mm diameter circular glass coverslips fitted in 24-well plates at a density of 2 × 10^4^ cells per coverslip and incubated at 37°C with 5% CO_2_/95% O_2_. The following day, the cells were treated with either 5-mM [Ca^2+^]_o_ or 10-μM NPS-R568 for 4 h, fixed with 4% paraformaldehyde for 15 min, and stored in 100% methanol at −20°C. A commercial Duolink^®^ proximity ligation assay (PLA) system (Merck) was used according to the manufacturer's protocol. The following antibodies were used: rabbit polyclonal anti-*KCNQ2* (ab22897, 1:200) and mouse monoclonal anti-phosphoserine conjugated to Duolink^®^ PLA MINUS probe (DUO87012, 1:50). An Olympus BX61 fluorescence microscope was used to capture three random areas per coverslip; the density of PLA signals (red fluorescent puncta/cell) were counted using ImageJ software.

### 2.8 Transient transfection of HEK-CaSR with *KCNQ2/3* cDNA

HEK-CaSR were plated on specialised silicone chambers as mentioned above and incubated at 37°C with 5% CO_2_/95% O_2_ overnight. Transfection was performed the following day. In an Eppendorf tube, 2 μL of human *KCNQ2/3* cDNA plasmid (0.9 g·L^−1^) and 1 μL of pmax green fluorescent protein vector (0.5 g·L^−1^) (Lonza, Cologne, Germany) were diluted in 125 μL of Opti-MEM medium (Life Technologies). In a separate tube, 5 μL of Lipofectamine™ 2000 transfection reagent (Life Technologies) was diluted in 125 μL of Opti-MEM^®^ medium and incubated at room temperature for 5 min. The diluted cDNA and diluted Lipofectamine™ 2000 transfection reagent were gently mixed then incubated at room temperature for a further 20 min. Approximately 35 μL of the mixture was added to each chamber and all chambers were incubated at 37°C with 5% CO_2_/95% O_2_. The culture medium in each chamber was replaced the next day. For the control group, *KCNQ2/3* cDNA was omitted.

### 2.9 Chronic incubation of hiPSC-derived nociceptive-like neurons with an algogenic cocktail and/or the calcilytic NPS-2143

To induce hyperexcitability of hiPSC-derived nociceptive-like neurons, a cocktail of algogenic mediators was prepared. The algogenic cocktail incorporated known Kv7 channel-suppressing algogenic mediators, including bradykinin (Liu et al., 2010), ATP (Zaika et al., 2007) and substance P (Linley et al., 2012). In addition, TNFα, a cytokine associated with neuropathic pain, was included (Bowen et al., 2006; Koizumi et al., 2021). These algogenic mediators were diluted in SCM2 medium (final concentration: 1-μM bradykinin, 1-μM substance P, 10-μM ATP and 50-ng·mL^−1^ TNFα) and applied in culture for three to 4 days.

To investigate the effect of a calcilytic on excitability, 1 μM NPS-2143 was applied both alone and in combination with the algogenic cocktail to separate cultures of hiPSC-derived nociceptive-like neurons for 3 to 4 days. Analyses of the spontaneous activity, iAP type, characteristics of the first iAP and spike frequency were performed.

### 2.10 Data and statistical analyses

All data are presented as mean ± standard error of the mean (SEM). Data were analysed and visualised using Clampfit 10.2 (Molecular Devices) and GraphPad Prism 9 (GraphPad Software, San Diego, USA).

For the analysis of concentration-response experiments, the voltage change induced by different concentration of [Ca^2+^]_o_ relative to 0.1-mM [Ca^2+^]_o_ was fitted with the Hill equation:

$$\text{Y}=\;\text{Bottom}\;+\frac{\text{Top}-\text{Bottom}\;}{1+{\left(\frac{\;\text{EC}50\;}{\text{X}}\right)}^{\text{H}}}$$

where *Y* is the voltage change, *X* is the [Ca^2+^]_o_, EC_50_ is the half maximal depolarisation response and *H* is Hill slope.

The Shapiro–Wilk test was used to assess normality of the data. Statistical tests (chi-squared test, one-sample *t* test, Wilcoxon signed rank test, paired *t* test, unpaired *t* test, Mann–Whitney U test, one-way analysis of variance (ANOVA), two-way ANOVA and Kruskal-Wallis test as stated in text and figure legends) were selected based on normality of the data and carried out in GraphPad Prism 9; statistical significance differences were recognised at *P* < 0.05 throughout the study.

### 2.11 Nomenclature of targets and ligands

Key protein targets and ligands in this article are hyperlinked to corresponding entries in https://www.guidetopharmacology.org and are permanently archived in the Concise Guide to PHARMACOLOGY2021/22 (Alexander, Christopoulos, et al., 2021; Alexander, Kelly, et al., 2021; Alexander, Mathie, et al., 2021).

## 3 Results

### 3.1 Activation of CaSR attenuates *I*_m_ and causes depolarisation through neuronal Kv7.2/7.3 channels

**Figure 1:**
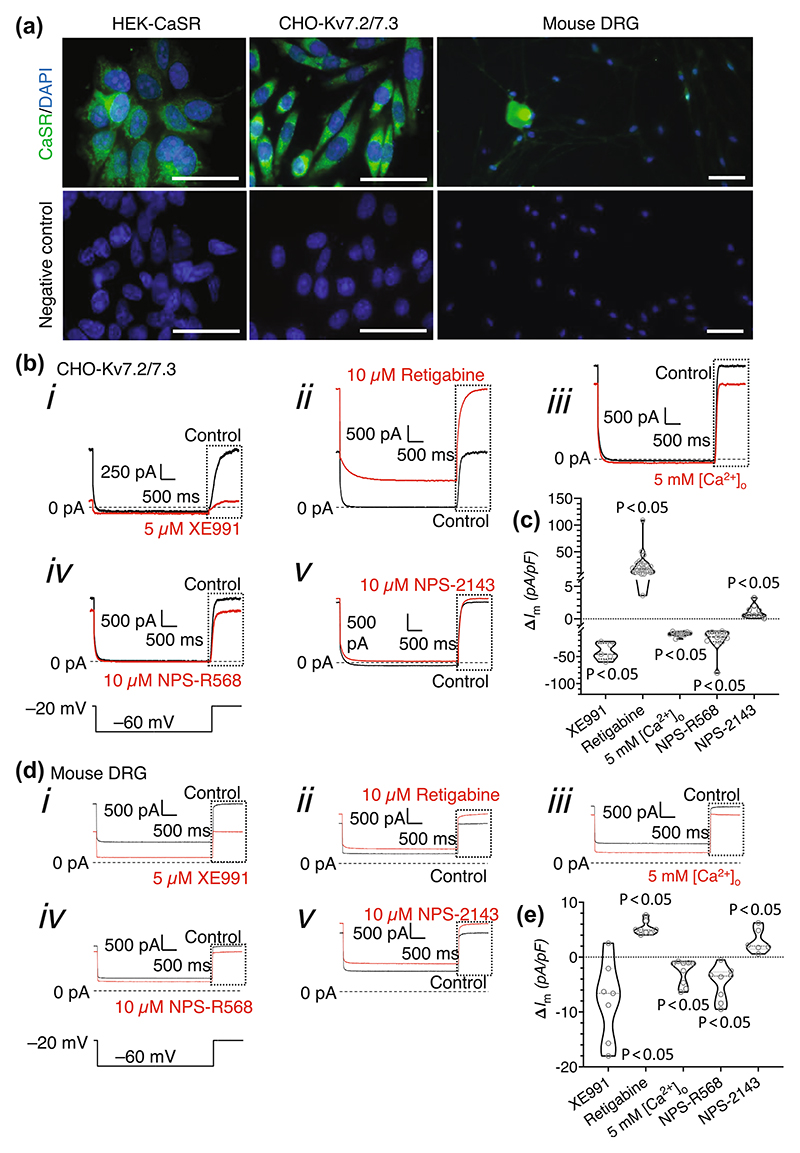
Effect of Calcium-sensing receptor (CaSR) modulation on *I*_m_. (a) Immunofluorescence images show CaSR (green) expressed in CHO-Kv7.2/7.3, HEK-CaSR (ab19347 at 1:100 dilution and A21202 at 1:200) and mouse dorsal root ganglia (DRG) (SAB4503369 at 1:100 dilution and A21206 at 1:200 dilution). Expression of CaSR is much more pronounced in mouse DRG neurons than in glial cells. Images were obtained with Olympus BX61 fluorescence microscope and are representative of three independent experiments. Scale bars: 50 μm. (b) Exemplar traces show the *I*_m_ of CHO-Kv7.2/7.3 in control (0.5-mM [Ca^2+^]_o_) and in the presence of 5-μM XE991 (i), 10-μM retigabine (ii), 5-mM [Ca^2+^]_o_ (iii), 10-μM NPS-R568 (iv) and 10-μM NPS-2143 (v). The voltage-stepped deactivation protocol and dotted outlines indicating the region of *I*_m_ quantification also are shown. (c) Graph shows the magnitude of *I*_m_ density change induced by 5-μM XE991 (n = 5 cells), 10-μM retigabine (n = 24 cells), 5-mM [Ca^2+^]_o_ (n = 12 cells), 10-μM NPS-R568 (n = 12 cells) and 10-μM NPS-2143 (n = 9 cells) in CHO-Kv7.2/7.3. (d) Exemplar traces show the *I*_m_ of mouse DRG neurons in control (0.5-mM [Ca^2+^]_o_) and in the presence of 5-μM XE991 (i), 10-μM retigabine (ii), 5-mM [Ca^2+^]_o_ (iii), 10-μM NPS-R568 (iv) and 10-μM NPS-2143 (v). The voltage-stepped deactivation protocol and dotted outlines indicating the region of *I*_m_ quantification also are shown. (e) Graph shows the magnitude of *I*_m_ density change induced by 5-μM XE991 (n = 7 cells), 10-μM retigabine (n = 8 cells), 5-M [Ca^2+^]_o_ (n = 9 cells), 10-μM NPS-R568 (n = 8 cells) and 10-μM NPS-2143 (n = 6 cells) in mouse DRG neurons. Data are shown as means±SEM. For CHO-Kv7.2/7.3, statistical analyses were performed using a two-tailed one-sample *t*-test to a theoretical mean of 0 (C: XE991, 5-mM [Ca^2+^]_o_ and NPS-2143) and the Wilcoxon signed rank test (C: retigabine and NPS-R568). For mouse DRG neurons, statistical analyses were performed using two-tailed one-sample *t*-test except for the effect of 5-mM [Ca^2+^]_o_, in which the Wilcoxon signed rank test was used.

The functional crosslink between the CaSR and Kv7.2/7.3 channels was investigated using several cellular models: CHO-Kv7.2/7.3, HEK-CaSR, mouse DRG neurons and hiPSC-derived nociceptive-like neurons. Molecular expression of CaSR in CHO-Kv7.2/7.3 and mouse DRG was verified with immunocytochemistry; HEK-CaSR were used as a positive control (Figure 1a).

Using the voltage-stepped deactivation protocol, the effects of high [Ca^2+^]_o_ (5 mM) and NPS-R568, a PAM of CaSR, on *I*_m_ of CHO-Kv7.2/7.3 were examined in the whole-cell configuration. CHO-Kv7.2/7.3 exhibited an outward *I*_m_ of 43.7 ± 4.8 pA/pF (n = 29 cells) when bathed in a standard solution (0.5-mM [Ca^2+^]_o_). Application of 5-μM XE991, a Kv7 channel blocker, or 10-μM retigabine, a Kv7 channel opener, significantly decreased by −41.1 ± 6.9 pA/pF (−99.8 ± 0.2%; P=0.004), or increased by 23.2 ± 4.4 pA/pF (61.8 ± 9.7%; P=0.0001) the *I*_m_ of CHO-Kv7.2/7.3, respectively. In a similar fashion to XE991, high [Ca^2+^]_o_ and 10-μM NPS-R568 significantly reduced the *I*_m_ of CHO-Kv7.2/7.3 by −8.0 ± 1.1 pA/pF (P=0.0001) and −18.7 ± 6.1 pA/pF (P=0.001), respectively. A CaSR NAM, 10-μM NPS-2143, significantly increased *I*_m_ of CHO-Kv7.2/7.3 by 1.1 ± 0.3 pA/pF (P=0.009; Figure 1b,c). In mouse DRG neurons, 5-μM XE991 significantly reduced *I*_m_ by −7.9 ± 2.7 pA/pF (−163.0 ± 67.3%; P=0.028). However, a clear heterogeneity in mouse DRG neurons with respect to the XE991-sensitive *I*_m_ was recognised. Retigabine (10 μM) significantly enhanced *I*_m_ by 5.3 ± 0.4 pA/pF (140.5 ± 22.9%; P=0.001). High [Ca^2+^]_o_ and 10-μM NPS-R568 significantly reduced *I*_m_ of mouse DRG neurons by −2.7 ± 0.8 pA/pF (P=0.004) and −4.8 ± 1.1 pA/pF (0.004), respectively. In addition, a significant effect of 10-μM NPS-2143 in enhancing the *I*_m_ (2.9 ± 0.9 pA/pF; P=0.021) was observed (Figure 1d,e).

**Figure 2:**
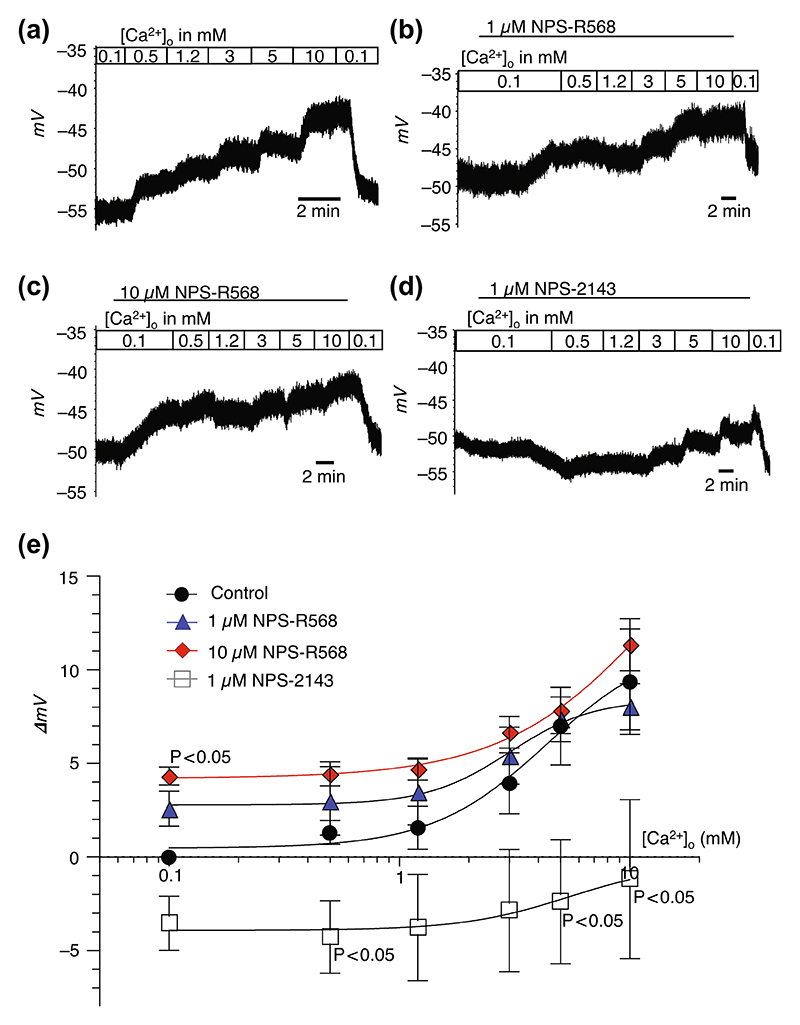
Effect of CaSR modulation on the RMP. (a–d) Exemplar traces show the effects of [Ca^2+^]_o_ (0.1, 0.5, 1.2, 3, 5 and 10 mM) on the resting membrane potential (RMP) of CHO-Kv7.2/7.3 in the control condition (a), 1-μM NPS-R568 (b), 10-μM NPS-R568 (c) and 1-μM NPS-2143 (d). (e) Fitted concentration-response curves in each condition are shown (n = 5 cells per condition). Data are shown as means ± SEM. One-sample *t*-test to a theoretical mean of 0 was used to test the effects of 1-μM NPS-R568, 10-μM NPS-R568 and 1-μM NPS-2143 on the RMP in the presence of 0.1-mM [Ca^2+^]_o_, and one-way analysis of variance (ANOVA) with the Dunnett’s multiple comparison test was used to compare the effects of 1-μM NPS-R568, 10-μM NPS-R568 and 1-μM NPS-2143 on the RMP to the control in the presence of 0.5– 10-mM [Ca^2+^]_o_. Where *P* is not provided, no significant difference was observed.

The effect of CaSR on the RMP of CHO-Kv7.2/7.3 was investigated. Administration of [Ca^2+^]_o_ (0.1 to 10 mM) caused depolarisation in a concentration-dependent manner with an EC_50_ value of 4.3 mM (Figure 2a,e and Table S1). The relationship between [Ca^2+^]_o_ and voltage change was then investigated in the presence of NPS-R568 at two concentrations (1 and 10 μM) (Figure 2b,c); the concentration-dependent effect of [Ca^2+^]_o_ on depolarisation was preserved in the presence of NPS-R568 (Figure 2e). Administration of 1-μM NPS-2143, a NAM or calcilytic, caused a hyperpolarising shift of the [Ca^2+^]_o_-voltage change response (Figure 2d,e). The degree of depolarisation by 0.5-, 5- and 10-mM [Ca^2+^]_o_ in the presence of NPS-2143 was significantly decreased, compared with the control (P=0.033, P=0.018 and P=0.037, respectively; Figure 2e).

**Figure 3:**
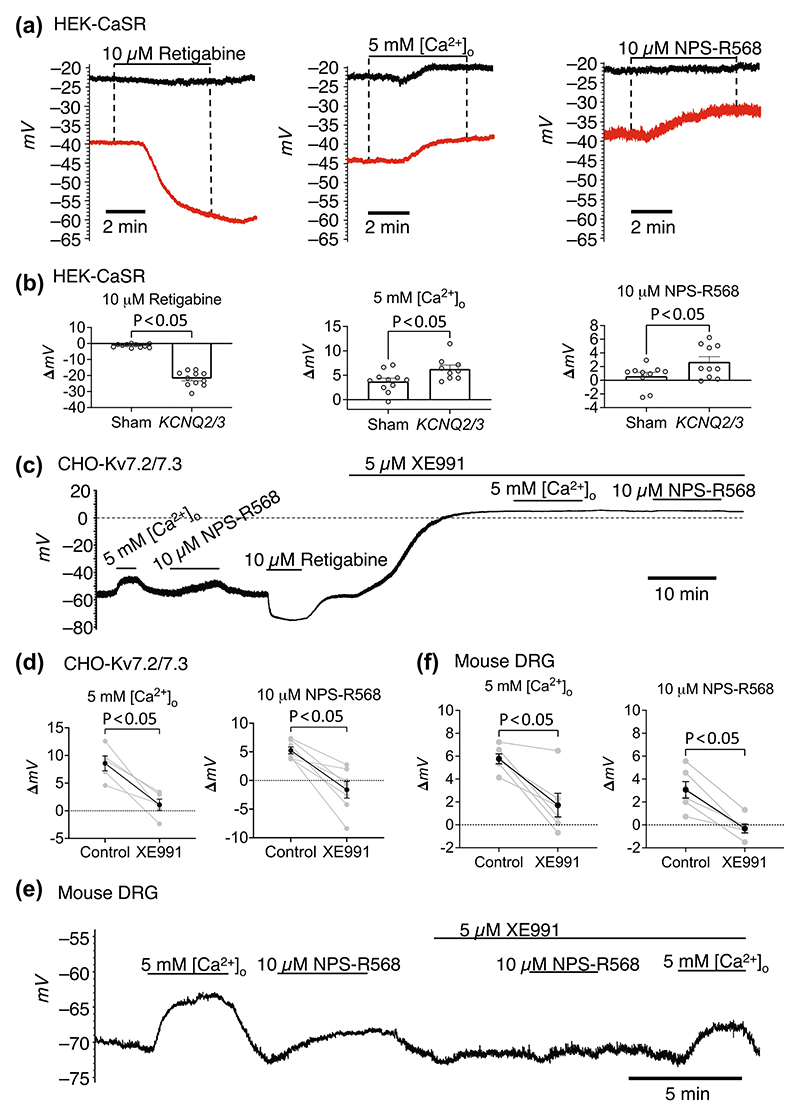
Molecular and pharmacological identification of the functional crosslink between CaSR and Kv7.2/7.3 channels. (a) Exemplar traces show the effects of 10-μM retigabine, 5-mM [Ca^2+^]_o_ and 10-μM NPS-R568 on the resting membrane potential (RMP) of sham-transfected HEK-CaSR (black traces) and HEK-CaSR transiently transfected with *KCNQ2/3* cDNA (red traces). (b) Graphs compare the magnitude of hyperpolarisation by 10-μM retigabine (n = 11 cells) and depolarisation by 5-mM [Ca^2+^]_o_ (n = 9–11 cells) and 10-μM NPS-R568 (n = 10 cells) between sham-transfected and *KCNQ2/3-*transfected HEK-CaSR. (c) Exemplar trace shows the effects of 5-mM [Ca^2+^]_o_ and 10-μM NPS-R568 on the RMP of CHO-Kv7.2/7.3 in the control condition and in the presence of 5-μM XE991. (d) Graphs compare the magnitude of depolarisation by 5-mM [Ca^2+^]_o_ (n = 5 cells) and 10-μM NPS-R568 (n = 7 cells) before and after the application of 5-μM XE991 in CHO-Kv7.2/7.3. (e) Exemplar trace shows the effects of 5-mM [Ca^2+^]_o_ and 10-μM NPS-R568 on the RMP of a mouse DRG neuron in the control condition and in the presence of 5-μM XE991. (f) Graphs compare the magnitude of depolarisation by 5-mM [Ca^2+^]_o_ and 10-μM NPS-R568 before and after the application of 5-μM XE991 in mouse DRG neurons (n = 6 cells). Data are shown as means ± SEM. Statistical analyses were performed using a two-tailed unpaired *t*-test (b) and two-tailed paired *t*-test (d and f).

To test whether the observed effect of t CaSR on the RMP was mediated through Kv7.2/7.3 channels, transient transfection of HEK-CaSR with a *KCNQ2/3* cDNA plasmid was performed, and the depolarising effect of CaSR activation was investigated. In sham-transfected HEK-CaSR, 10-μM retigabine and 10-μM NPS-R568 did not induce a significant change in the RMP, while depolarisation by 5-mM [Ca^2+^]_o_ (3.8 ± 0.7 mV) was observed. In *KCNQ2/3* cDNA-transfected cells, retigabine induced significant hyperpolarisation (−21.9 ± 1.4 mV; P=0.0001), confirming the functional expression of Kv7.2/7.3 channels. The magnitude of depolarisation by 5-mM [Ca^2+^]_o_ and 10-μM NPS-R568 was also significantly increased by 2.6 ± 1.0 mV (P=0.021) and 2.1 ± 0.9 mV (P=0.037), respectively (Figure 3a,b). These data suggest that CaSR-mediated depolarisation is influenced by the expression of Kv7.2/7.3 channels.

Further evidence to support the CaSR-Kv7.2/7.3 channel crosslink was derived from experiments comparing the magnitude of depolarisation induced by CaSR activation before and after the application of 5-μM XE991. Increasing [Ca^2+^]_o_ from 0.5 to 5 mM and application of 10-μM NPS-R568 in the presence of 0.5-mM [Ca^2+^]_o_ caused depolarisation in CHO-Kv7.2/7.3 and mouse DRG neurons. Application of XE991 depolarised CHO-Kv7.2/7.3 and significantly attenuated the depolarising effects of 5-mM [Ca^2+^]_o_ by −7.5 ± 1.5 mV (P=0.008) and NPS-R568 by −6.9 ± 1.4 mV (P=0.0026; Figure 3c,d). In mouse DRG neurons, application of 5-mM [Ca^2+^]_o_ and NPS-R568 significantly depolarised the neurons by 5.8 ± 0.4 mV (P=0.006) and 3.1 ± 0.7 mV (P=0.002), respectively. Application of XE991 did not alter the RMP of mouse DRG neurons, but significantly attenuated the depolarising effects of 5 mM [Ca^2+^]_o_ by −4.1 ± 0.9 mV and NPS-R568 by −3.4 ± 0.6 mV (Figure 3e,f).

**Figure 4:**
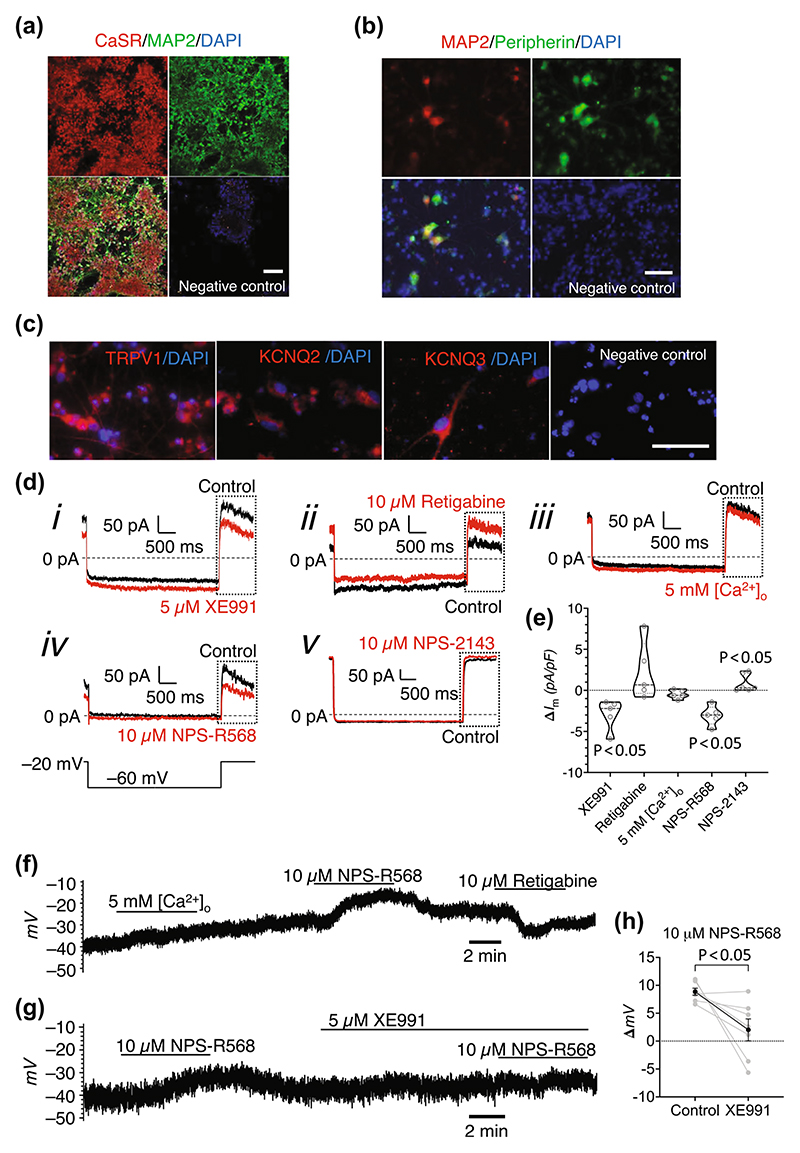
Characterisation of hiPSC-derived neurons and verification of the CaSR-Kv7 channel crosslink. (a) Immunofluorescence images show CaSR (red; SAB4503369 at 1:100 dilution and A21207 at 1:250 dilution) and MAP 2 (green; M1406 at 1:100 dilution and ab1510117 at 1:250 dilution) expressed in hiPSC-derived neurons. Images were obtained with Zeiss LSM800 laser confocal scanning microscope and are representative of three independent experiments. Scale bars: 100 μm. (b) Immunofluorescence image shows MAP 2 (red; M1406 at 1:250 dilution and A21203 at 1:500 dilution) and peripherin (green; ab4666 at 1:250 dilution and A21206 at 1:500 dilution) expressed in hiPSC-derived neurons. Images were obtained with Olympus BX61 fluorescence microscope and are representative of three independent experiments. Scale bars: 50 μm. (c) Immunofluorescence image shows TRPV1 (red; ab3487 at 1:100 dilution and A21207 at 1:200 dilution), Kv7.2 channels (red; ab22897 at 1:200 dilution and A21207 at 1:200 dilution) and Kv7.3 channels (red; PA1–930 at 1:100 dilution and A21207 at 1:200 dilution) expressed in hiPSC-derived neurons. Images were obtained with Olympus BX61 fluorescence microscope and are representative of three independent experiments. Scale bars: 50 μm. (d) Exemplar traces show *I*_m_ of hiPSC-derived nociceptive-like neurons in control (0.5-mM [Ca^2+^]_o_) and in the presence of 5-μM XE991 (i), 10-μM retigabine (ii), 5-mM [Ca^2+^]_o_ (iii), 10-μM NPS-R568 (iv) and 10-μM NPS-2143 (v). The voltage-stepped deactivation protocol and dotted outlines indicating the region of *I*_m_ quantification also are shown. (e) Graph shows the magnitude of *I*_m_ density change induced by 5-μM XE991, 10-μM retigabine, 5-mM [Ca^2+^]_o_, 10-μM NPS-R568 and 10-μM NPS-2143 in hiPSC-derived nociceptive-like neurons (n = 5–6 cells). (f) Exemplar trace shows the effects of 5-mM [Ca^2+^]_o_, 10-μM NPS-R568 and 10-μM retigabine on the resting membrane potential (RMP) of a hiPSC-derived nociceptive-like neurons. (g) Exemplar trace shows the effect of 10-μM NPS-R568 on the RMP of a hiPSC-derived nociceptive-like neurons before and after the application of 5-mM XE991. (H) Graph compares the magnitude of depolarisation by 10-μM NPS-R568 before and after the application of 5-μM XE991 in hiPSC-derived nociceptive-like neurons (n = 7 cells). Data are shown as means ± SEM. Statistical analyses were performed using a two-tailed one-sample *t*-test to a theoretical mean of 0 (e: XE991, retigabine, 5-mM [Ca^2+^]_o_ and NPS-R568), the Wilcoxon signed rank test (e: NPS-2143) and a two-tailed paired *t*-test (h).

The effect of CaSR activation on *I*_m_ and RMP was then investigated in nociceptive-like neurons, which were differentiated from HD33n1 hiPSCs. Immunocytochemistry confirmed the expression of CaSR, a neuronal marker MAP2, a peripheral neuronal marker peripherin (Figure 4a,b), a nociceptive marker TRPV1, and Kv7.2/7.3 channels (Figure 4c). In a standard solution, hiPSC-derived nociceptive-like neurons had the outward *I*_m_ of 5.0 ± 0.7 pA/pF (n = 16 cells). Application of 5-μM XE991 significantly reduced the *I*_m_ by −2.9 ± 0.8 pA/pF (−76.3 ± 43.1%; P=0.024). No significant effect of either 10-μM retigabine or 5-mM [Ca^2+^]_o_ on *I*_m_ was observed. Application of 10-μM NPS-R568 significantly reduced *I*_m_ by −3.1 ± 0.5 pA/pF (P=0.005), whereas 10-μM NPS-2143 minimally yet significantly increased *I*_m_ by 0.9 ± 0.4 pA/pF (P=0.031; Figure 4d,e). The effect of 5-mM [Ca^2+^]_o_ on the RMP varied between cells, whereas a consistent depolarising effect of 10-μM NPS-R568 was observed (Figure 3f). In the presence of 5-μM XE991 however, NPS-R568-induced depolarisation was significantly attenuated by −6.8 ± 2.5 mV (P=0.032) in hiPSC-derived nociceptive-like neurons (Figure 4g,h).

### 3.2 CaSR-mediated depolarisation of CHO-Kv7.2/7.3 is sensitive to 8-Br-cAMP but not to NiCl_2_, BAPTA-AM and U73122

**Figure 5:**
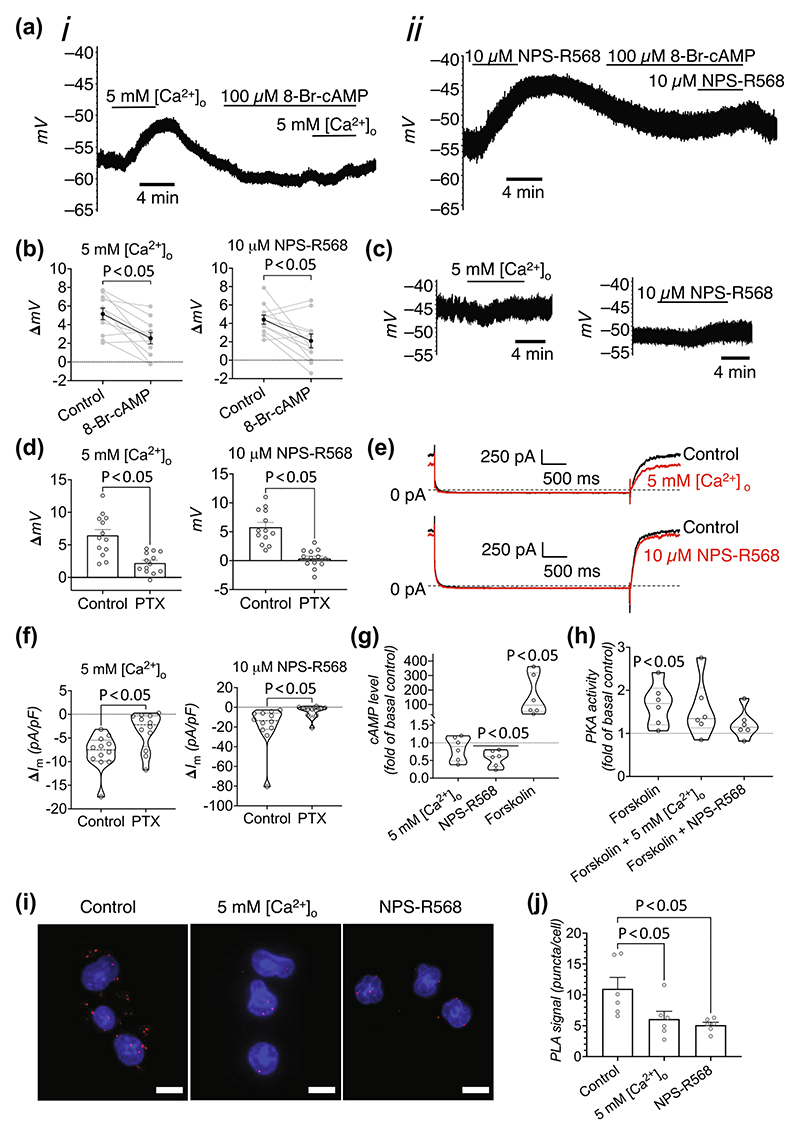
CaSR-Kv7.2/7.3 channel crosslink is mediated through G_i/o_ protein-AC-cAMP-PKA signalling. (a) Exemplar traces show the effects of 100-μM 8-Br-cAMP on depolarisation by 5-mM [Ca^2+^]_o_ (i) and 10-μM NPS-R568 (ii) in CHO-Kv7.2/7.3. (b) Graphs compare the magnitude of depolarisation by 5-mM [Ca^2+^]_o_ and 10-μM NPS-R568 before and after the application of 8-Br-cAMP (n = 11 cells) in CHO-Kv7.2/7.3. (c) Exemplar traces show the effects of 5-mM [Ca^2+^]_o_ and 10-μM NPS-R568 on the RMP of CHO-Kv7.2/7.3 after 20–48 h of 100-ng·mL^−1^ PTX incubation. (d) Graphs compare the magnitude of depolarisation by 5-mM [Ca^2+^]_o_ and 10-μM NPS-R568 between control and PTX-treated CHO-Kv7.2/7.3 (n = 13 cells). (e) Exemplar traces show *I*_m_ of PTX-treated CHO-Kv7.2/7.3 in the control solution and in the presence of 5-mM [Ca^2+^]_o_ and 10-μM NPS-R568. (f) Graphs compares the magnitude of *I*_m_ density reduction induced by 5-mM [Ca^2+^]_o_ and 10-μM NPS-R568 between control and PTX-treated CHO-Kv7.2/7.3 (n = 12 cells). (g) Graph shows the fold change of intracellular cAMP levels induced by 5-mM [Ca^2+^]_o_, 10-μM NPS-R568 and 10-μM forskolin in CHO-Kv7.2/7.3 (n = 6 experiments). (h) Graph shows the fold change of PKA activity induced by 10-μM forskolin, 10-μM forskolin plus 5-mM [Ca^2+^]_o_ and 10-μM forskolin plus 10-μM NPS-R568 in CHO-KV7.2/7.3 (n = 6 experiments). (i) Immunofluorescence images show phosphorylated serine-*KCNQ2* PLA signals of CHO-Kv7.2/7.3 in the control condition, 5-mM [Ca^2+^]_o_ and 10-μM NPS-R568. (j) Graph compares the density of PLA signals in CHO-Kv7.2/7.3 between three conditions: the control condition, 5-mM [Ca^2+^]_o_ and 10-μM NPS-R568 (n = 6 experiments). Data are shown as means ± SEM. Statistical analyses were performed using a two-tailed paired *t*-test (b), a two-tailed unpaired *t*-test (d), the Mann–Whitney U test (f), the Wilcoxon signed rank test to a theoretical mean of 1.0 (g and h) and one-way ANOVA with Dunnett's multiple comparison test to the control (j). Where *P* is not provided, no significant difference was observed.

To explore the potential molecular pathways linking CaSR and Kv7.2/7.3 channels, compounds targeting certain canonical G protein pathways following CaSR activation were used. The effects of 5-mM [Ca^2+^]_o_ and 10-μM NPS-R568 on the RMP of CHO-Kv7.2/7.3 were investigated before and after the application of each compound. The depolarising effects of 5-mM [Ca^2+^]_o_ and 10-μM NPS-R568 were significantly attenuated by −2.6 ± 0.7 mV (P=0.0028) and −2.3 ± 0.8 mV (P=0.017), respectively, in the presence of 100-μM 8-Br-cAMP, a PKA activator (Figure 5a,b). In contrast, a non-selective blocker of Ca^2+^ influx NiCl_2_, a chelator of [Ca^2+^]_i_ BAPTA-AM and a PLC inhibitor U73122 had no effect on CaSR-mediated depolarisation (Figure S1a–d).

### 3.3 CaSR-mediated depolarisation and *I*_m_ reduction of CHO-Kv7.2/7.3 are sensitive to pertussis toxin

The sensitivity of CaSR-mediated depolarisation to PKA activation suggested the involvement of the G_i/o_ protein pathway. To verify this assumption, the effect of 100-ng·mL^−1^ PTX, a G_i/o_ protein inhibitor, on the effects of high [Ca^2+^]_o_ and NPS-R568 in CHO-Kv7.2/7.3 was investigated. The PTX-treated cells exhibited significantly reduced depolarisation induced by both 5-mM [Ca^2+^]_o_ (mean difference: −4.3 ± 1.0 mV; P=0.0002) and 10 μM NPS-R568 (mean difference: −5.4 ± 0.9 mV; P=0.0001) (Figure 5c,d). The effects of 5 mM [Ca^2+^]_o_ and 10 μM NPS-R568 on *I*_m_ density in PTX-treated cells were significantly attenuated by 4.5 ± 1.6 pA/pF (P=0.008) and 13.9 ± 6.4 pA/ pF (P=0.003), respectively (Figure 5e,f).

### 3.4 Activation of CaSR reduces basal intracellular cAMP levels, forskolin-stimulated PKA activity and serine phosphorylation in CHO-Kv7.2/7.3

Coupling of CaSR to the G_i/o_ protein pathway was further confirmed by investigating the effects of CaSR activation on intracellular cAMP levels and PKA activity in CHO-Kv7.2/7.3. Application of 10 μM NPS-R568 significantly reduced basal intracellular cAMP levels by 0.44 ± 0.1 fold (P=0.031), whereas 10-μM forskolin, a transmembrane AC activator, increased intracellular cAMP levels by 158.6 ± 57.2 fold (P=0.031; Figure 5g). In addition, forskolin significantly increased PKA activity by 1.7 ± 0.2 fold (P=0.031); such significance was not apparent when forskolin was applied in combination with either 5 mM [Ca^2+^]_o_ or NPS-R568 (Figure 5h).

The effect of CaSR activation on serine phosphorylation of the Kv7.2 channel subunit was investigated using the PLA. High [Ca^2+^]_o_ and 10-μM NPS-R568 significantly reduced the density of PLA signals by −4.9 ± 1.9 punctae/cell (P=0.034) and −5.9 ± 1.9 punctae/cell (P=0.012), respectively, in CHO-Kv7.2/7.3 (Figure 5i,j). No PLA signal was observed in negative controls where primary antibodies were omitted.

Following characterisation of the CaSR-Kv7 channel crosslink in several cellular models, our next series of experiments explored the possibility of targeting such crosslink for controlling neuronal hyperexcitability using the hiPSC-derived hyperexcitability model due to its scalability and being a human-derived model.

### 3.5 An algogenic cocktail enhances induced excitability of hiPSC-derived nociceptive-like neurons without affecting spontaneous activity

**Figure 6:**
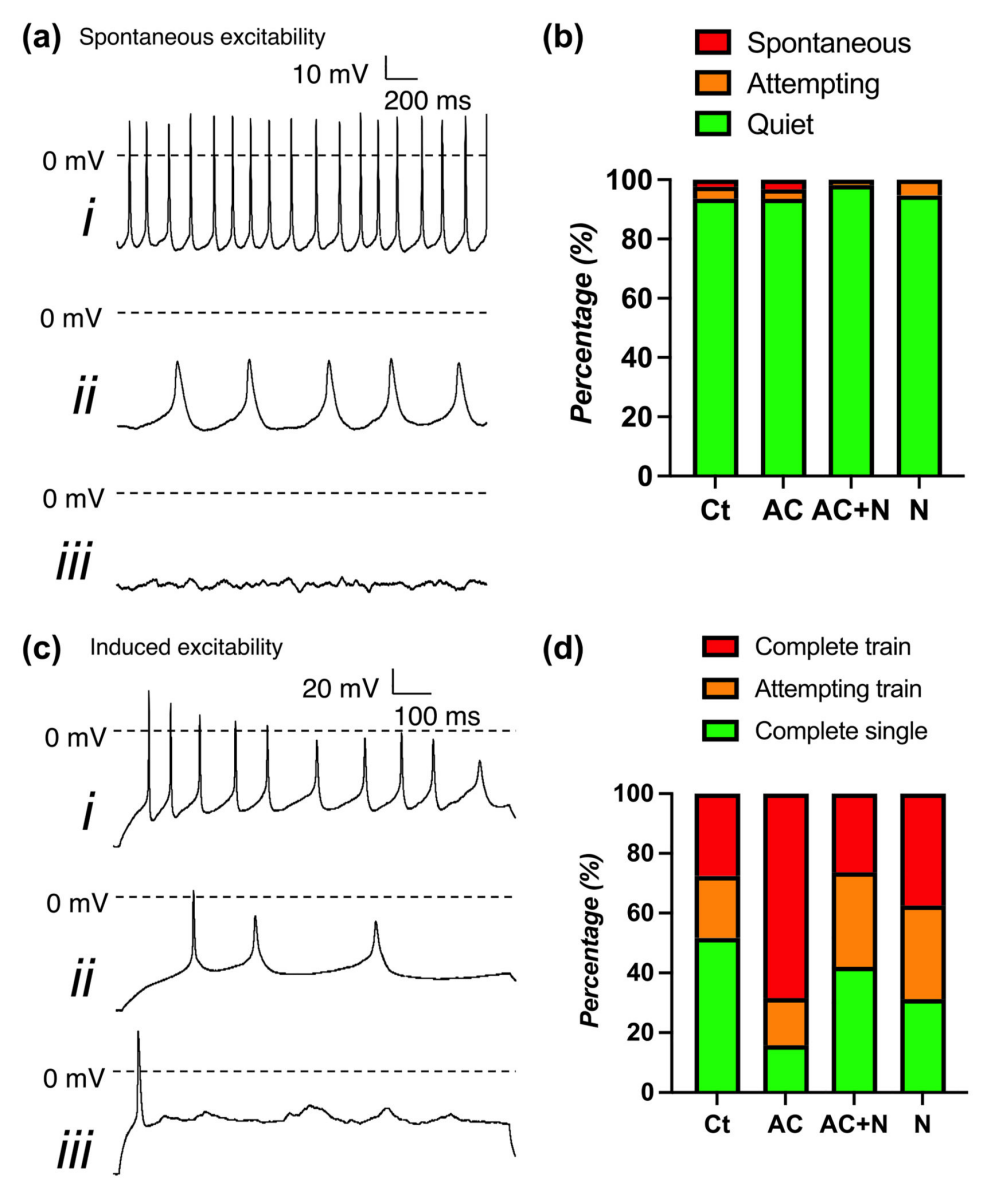
Effects of an algogenic cocktail and NPS-2143 on the spontaneous and induced excitability of hiPSC-derived nociceptive-like neurons. (a) hiPSC-derived nociceptive-like neurons were classified as having complete sAP (i) when at least one AP with its peak above 0 mV was observed. Attempting sAP (ii) was defined when an abrupt change in the RMP that looked like an AP occurred but did not reach 0 mV. When no AP was present during a 1-min period, the neurons were classified as quiet (iii). (b) Graph shows the percentage of hiPSC-derived nociceptive-like neurons at each spontaneous excitability level in four conditions: control (Ct; n = 125 cells from nine cultures), the algogenic cocktail (AC; n = 62 cells from three cultures), the algogenic cocktail plus 1-μM NPS-2143 (AC + N; n = 56 cells from four cultures) and 1-μM NPS-2143 (N; n = 38 cells from three cultures). (c) hiPSC-derived nociceptive-like neurons were classified as having a complete train of iAPs when more than one iAP overshot above 0 mV (i). When multiple iAPs were observed but only one iAP overshot above 0 mV, the cells were classified as having an attempting train of iAPs (ii). Cells with a single iAP overshooting above 0 mV were defined as having a complete single iAP (iii). (d) Graph shows the percentage of hiPSC-derived nociceptive-like neurons at each induced excitability level in four conditions: control (Ct; n = 29 cells from seven cultures), the algogenic cocktail (AC; n = 19 cells from three cultures), the algogenic cocktail plus 1-μM NPS-2143 (AC + N; n = 19 cells from four cultures) and 1-μM NPS-2143 (N; n = 16 cells from three cultures). Statistical analyses were performed using the chi-squared test.

**Figure 7:**
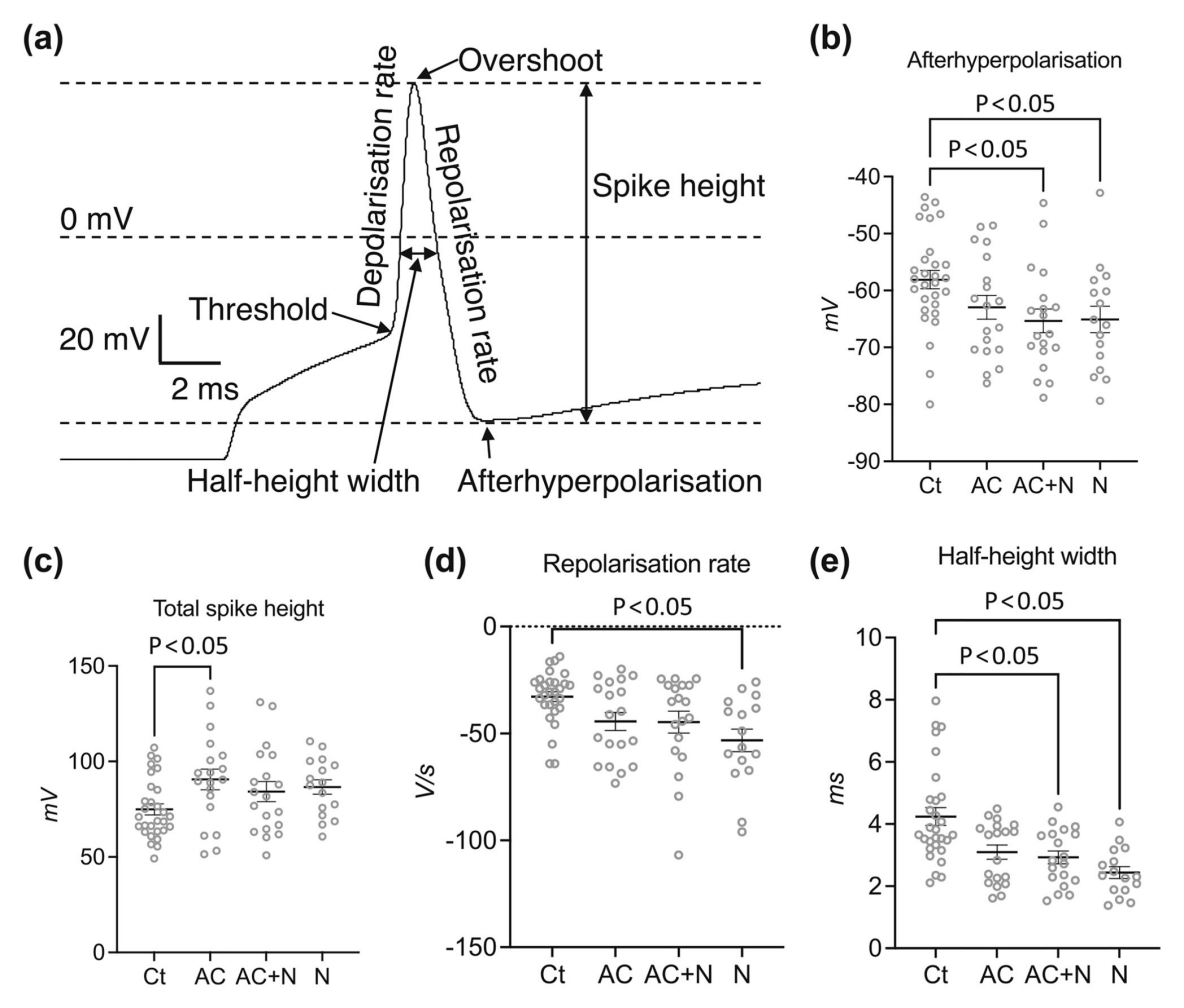
Effects of the algogenic cocktail and NPS-2143 on the characteristics of the first iAP of hiPSC-derived nociceptive-like neurons. (a) Exemplar trace illustrates the seven characteristics of an iAP investigated: threshold, overshoot, afterhyperpolarisation, total spike height, depolarisation rate, repolarisation rate and half-height width. (b–e) Graphs compare afterhyperpolarisation (, total spike height, repolarisation rate and half-height width of the first iAP between four conditions: control (Ct; n = 29 cells from seven cultures), the algogenic cocktail (AC; n = 19 cells from three cultures), the algogenic cocktail plus 1-μM NPS-2143 (AC + N; n = 19 cells from four cultures) and 1-μM NPS-2143 (N; n = 16 cells from three cultures). Data are shown as means ± SEM. Statistical analyses were performed using one-way ANOVA with Dunnett's multiple comparison test to the control (b and c) and the Kruskal–Wallis test with Dunn’s multiple comparison test to the control (d and e). Where *P* is not provided, no significant difference was observed.

Functional assessment with patch-clamp electrophysiology demonstrated a spectrum of spontaneous and induced excitability in the hiPSC-derived nociceptive-like neurons (Figure 6a–d). Following 3 to 4 days of incubation with an algogenic cocktail, most of the hiPSC-derived nociceptive-like neurons showed no sAP, which was similar to the controls (chi-squared [6]=3.7, P=0.72; Figure 6). The proportion of the algogenic cocktail-treated neurons with a complete train of iAPs, however, was more than twice relative to the control (24.5% vs. 11.9%) (chi-squared [6]=11.8, P=0.068; Figure 6d). In addition, spike analysis of the first iAP was performed in the hiPSC-derived nociceptive-like neurons (Figure 7a–e). The total spike height of the algogenic cocktail-treated neurons was significantly increased by 15.7 ± 5.7 mV, compared to the controls (Figure 7c).

### 3.6 NPS-2143 rescues hiPSC-derived nociceptive-like neurons from hyperexcitability induced by the algogenic cocktail

Like the algogenic cocktail, 1-μM NPS-2143 did not impose a significant effect on the spontaneous excitability of hiPSC-derived nociceptive-like neurons (Figure 6b). When NPS-2143 was applied simultaneously with the algogenic cocktail, a similar distribution of the neurons in each induced excitability classification to the controls was observed (Figure 6d).

Spike analysis of the first iAP showed that NPS-2143 enhanced certain characteristics associated with the repolarisation-hyperpolarisation phase of APs, including afterhyperpolarisation, that in the presence of algogenic cocktail + NPS-2134 and NPS-2134 alone, demonstrated significant difference from the control (P=0.02 and P=0.04, respectively; Figure 7b), repolarisation rate significantly decreased in the presence of NP-S2134 (P=0.003; Figure 7d) and half-height width ssignificantly decreased in the presence of algogenic cocktail + NP-S2134 and NP-S2134 alone (P=0.016 and P=0.001, respectively; Figure 7e). The effect of the algogenic cocktail in enhancing the total spike height was sgnificant (P=0.021), whereas that effect was not apparent in the presence of NPS-2143 (Figure 7c). No significant difference in the threshold, overshoot and depolarisation rate was noted between the four groups.

### 3.7 Algogenic cocktail alters the relationship between the injected current levels and spike frequency in hiPSC-derived nociceptive-like neurons

**Figure 8:**
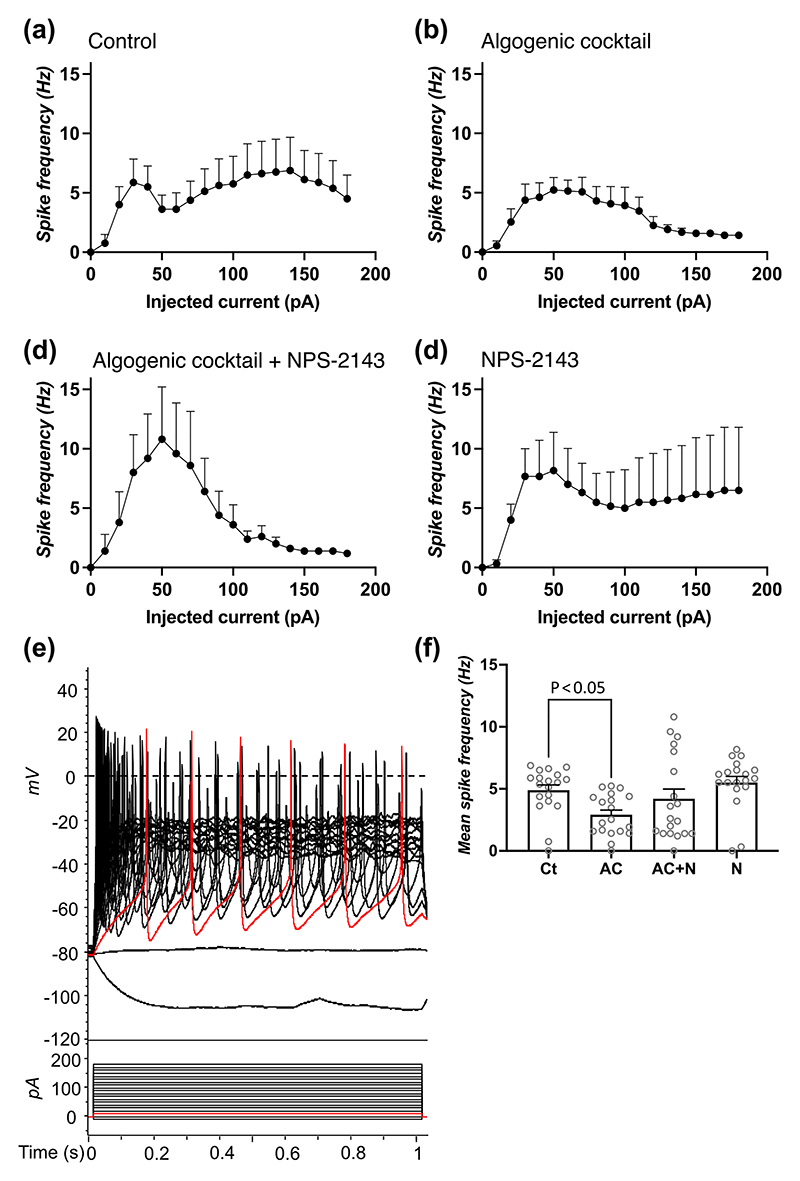
Effects of the algogenic cocktail and NPS-2143 on the iAP spike frequency of hiPSC-derived nociceptive-like neurons. (a–d) Graphs show the iAP spike frequency at each injected current level of hiPSC-derived nociceptive-like neurons expressing a complete train of iAPs in each condition. (e) Exemplar traces illustrate trains of induced action potentials in HD33n1 hiPSC-derived nociceptive-like neurons recorded in the current-clamp mode (upper panel) and the 1-s current injection protocol used for recording (lower panel). (f) Graphs compare the mean iAP spike frequency between four conditions: control (Ct; n = 8 cells from five cultures), the algogenic cocktail (AC; n = 13 cells from three cultures), the algogenic cocktail plus 1-μM NPS-2143 (AC + N; n = 5 cells from four cultures) and 1-μM NPS-2143 (N; n = 6 cells from three cultures). Data are shown as means±SEM. Statistical analysis was performed using two-way ANOVA with Dunnett's multiple comparison test to the control. Where *P* is not provided, no significant difference was observed.

The relationship between the injected current level and iAP spike frequency (the number of iAPs generated per second) of the hiPSC-derived nociceptive-like neurons exhibiting a complete train of iAPs was analysed in four different conditions (Figure 8a–f). Two-way ANOVA revealed significant effects of both the injected current level and condition on the spike frequency. In the controls, increasing the current amplitude correlated to a certain extent with an increase in the spike frequency, with two peaks observed at 30 pA and 140 pA (Figure 8a). In the algogenic cocktail-treated neurons, the spike frequency reached its peak at 50 pA then continuously declined with no secondary peak observed, so the mean spike frequency was significantly reduced compared to the Control (Figure 8b); a similar trajectory was found when the cells were treated in the algogenic cocktail plus NPS-2143 (Figure 8c). The NPS-2143 group showed a similar current-spike frequency relationship to the control, but the two peaks were slightly shifted to 50 and 170 pA, respectively (Figure 8d). Post hoc analysis with Dunnett's multiple comparison tests revealed the algogenic cocktail significantly reduced the mean iAP spike frequency (mean difference: –2.0 ± 0.6 Hz; P=0.002), which was rescued by co-treatment with NPS-2143 (Figure 8f).

## 4 Discussion

The significance of this study is two-fold: i) the discovery of the CaSR-Kv7.2/7.3 channel crosslink and ii) the therapeutic potential of targeting CaSR for neuronal hyperexcitability management.

### 4.1 Functional and molecular crosslink between CaSR and Kv7.2/7.3 channels

Our experiments on several cellular models demonstrated that CaSR activation by [Ca^2+^]_o_ and NPS-R568 reduced *I*_*m*_ and caused depolarisation, whereas inhibition by NPS-2143 increased *I*_*m*_. In the absence of Kv7.2/7.3 channel expression, or when the channels were blocked by XE991, CaSR-mediated depolarisation was nearly absent. Collectively, our findings suggest that CaSR activation causes depolarisation via Kv7.2/7.3 channel function inhibition. The allosteric modulators, NPS-R568 and NPS-2143, induced slower responses than high [Ca^2+^]_o_ in our study, which was to be expected and consistent with a previous report (Leong et al., 2021). The EC_50_ value for [Ca^2+^]_o_ could be underestimated as we did not test [Ca^2+^]_o_ at concentrations higher than 10 mM. Since inhibition of neuronal Kv7 channel activity leads to augmented excitability (Liu et al., 2010; Peng et al., 2017), our findings fit into the paradigm that CaSR activation enhances neuronal excitability (Vyleta & Smith, 2011) and it follows that CaSR inhibition with calcilytics may suppress neuronal hyperexcitability.

The lack of retigabine’s consistent effect on *I*_*m*_ and RMP in our hiPSC-derived model may suggest that a tonic activity of the CaSR suppresses the function of Kv7 channels. This notion is supported by a previous study, which showed that neurons isolated from CaSR-positive mice had more depolarised RMP and firing rates than those from CaSR-deficient mice (Martiszus et al., 2021), further supporting our theory that unlocking CaSR-mediated inhibition of Kv7 channels can be a strategy to control hyperexcitability. An alternative explanation is that our hiPSC-derived model may express a different Kv7 channel isoform that is less sensitive to retigabine (Schenzer et al., 2005). This possibility may explain the inconsistency concerning the effect of retigabine on *I*_*m*_ of our hiPSC-derived model and DRG neurons. The inconsistency in *I*_*m*_ modulation by high [Ca^2+^]_o_ implies that a higher concentration may be required for the hiPSC-derived model.

Our findings are in accordance with previous studies (Chang et al., 1998; Di Mise et al., 2018) in demonstrating that NPS-R568 reduced intracellular cAMP levels, confirming the coupling of CaSR to G_i/o_ protein. High [Ca^2+^]_o_, however, did not have a significant effect in our study, which could be explained by the preferential coupling of CaSR to G_q/11_ protein when CaSR is stimulated by [Ca^2+^]_o_, compared to NPS-R568 (Cook et al., 2015; Nemeth et al., 1998). G_q/11_ protein triggers inositol triphosphate (IP_3_)-induced [Ca^2+^]_i_ mobilisation, which can activate G_i/o_ protein-insensitive soluble AC and subsequently increases cAMP levels (Tresguerres et al., 2011), attenuating the overall response. Given our current understanding that cAMP signalling occurs in discrete intracellular microdomains (Tresguerres et al., 2011), the G_i/o_ protein-mediated cAMP reduction near the cell membrane can be masked by an increase in cAMP levels within the cytoplasmic microdomain when measured by ELISAs.

Since PKA function depends on cAMP levels (Søberg & Skålhegg, 2018), CaSR signalling may interfere with PKA activity. In our study, the forskolin-stimulated PKA activity was attenuated by NPS-R568 and to a lesser extent by high [Ca^2+^]_o_. Our findings thus confirm the association between CaSR activation and G_i/o_ protein-transmembrane AC-cAMP-PKA signalling. The smaller effect of [Ca^2+^]_o_ may pertain to the biased signalling of [Ca^2+^]_o_ towards the G_q/11_ protein pathway (Cook et al., 2015) and PKA being compartmentalised in a similar fashion to cAMP (Tresguerres et al., 2011).

**Figure 9:**
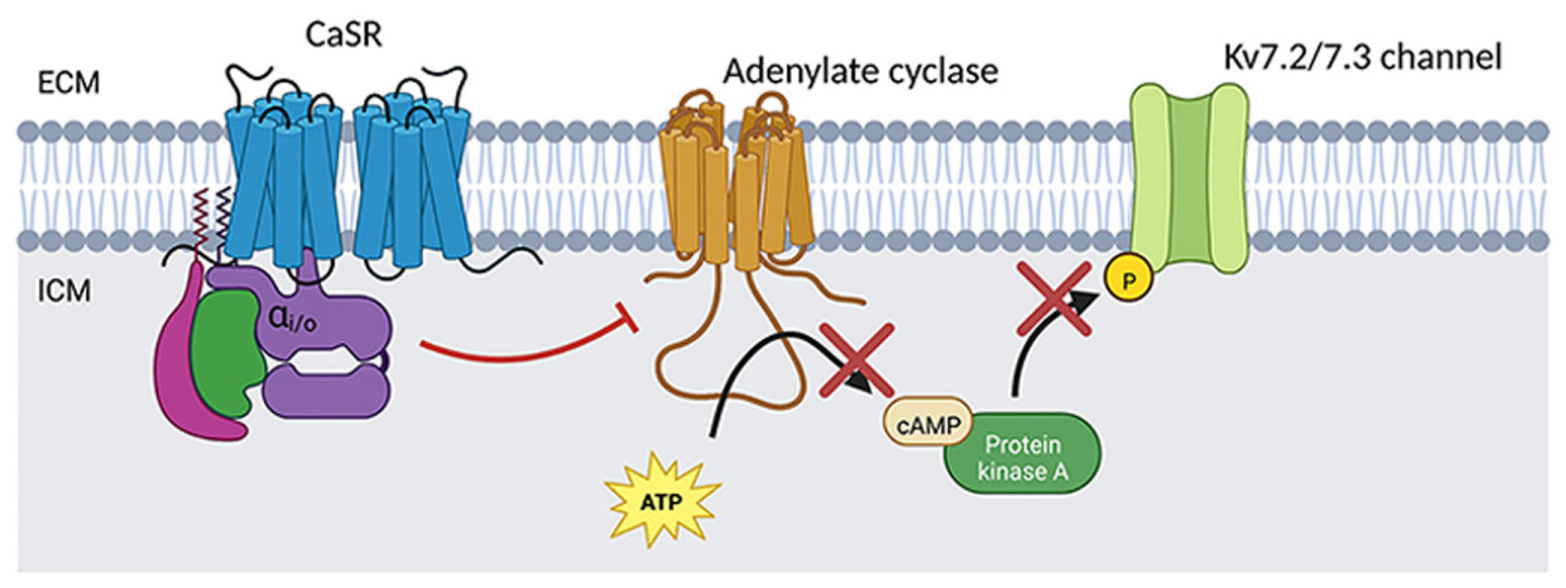
Proposed mechanism of the CaSR-Kv7.2/7.3 channel crosslink. Activation of CaSR stimulates G_i/o_ protein, which inhibits transmembrane AC. The results are decreases in cAMP levels, PKA activity, serine phosphorylation, and Kv7.2/7.3 channel function as represented by *I*_*m*_ reduction and depolarisation. CaSR, Ca^2+^-sensing receptor; ATP, adenosine triphosphate; cAMP, cyclic adenosine monophosphate; Kv7.2/7.3, voltage-gated K^+^ channel type 7.2/7.3; ECM, extracellular matrix; ICM, intracellular matrix. Created with BioRender.com.

A previous study has shown that *I*_*m*_ is potentiated by cAMP-dependent PKA phosphorylation at Ser^52^ of the Kv7.2 channel subunit (Schroeder et al., 1998), suggesting that a reduction in cAMP levels, PKA activity and serine phosphorylation would inhibit the Kv7.2/7.3 channel activity. In the present study, depolarisation and *I*_*m*_ reduction induced by high [Ca^2+^]_o_ and NPS-R568 were abated in PTX-treated CHO-Kv7.2/7.3. Furthermore, CaSR-mediated depolarisation was mitigated by 8-Br-cAMP. The PLA showed that CaSR activation significantly reduced phosphorylated serine signals associated with the Kv7.2 channel subunit. Altogether, our results suggest that G_i/o_ protein-transmembrane AC-cAMP-PKA signalling pathway mediates the CaSR-Kv7.2/7.3 channel crosslink (Figure 9).

### 4.2 Modulation of neuronal excitability via the CaSR-Kv7 channel crosslink

Unlike mouse DRG neurons (Figure S2), our hiPSC-derived nociceptive-like neurons demonstrated a variable iAP firing rate, with most generating a single iAP. Such a characteristic aligns with that of human sensory neurons (Davidson et al., 2014), highlighting interspecies differences in iAP firing.

Our data demonstrated that an algogenic cocktail induced hyper-excitability. Firstly, a higher percentage of the neurons with a complete train of iAPs was observed following algogenic cocktail treatment, possibly via Kv7.2/7.3 channel activity suppression (Passmore et al., 2003). However, the compliance of these neurons to generate iAPs also was reduced as a steady decrease in the iAP spike frequency at high injected current levels was found. A previous study showed that a more hyperpolarised Na_V_ current availability was associated with lower AP firing rates in hiPSC-derived neurons (Telezhkin et al., 2016). Hence, the algogenic cocktail likely induced a hyperpolarising shift of the Na_V_ current availability window, contributing to the observed reduction in the iAP spike frequency. Secondly, the total iAP spike height increased following algogenic cocktail treatment, which is similar to what was previously reported in hiPSC-derived nociceptive neurons of persistent pain patients and might suggest that Na_V_1.7 and Na_V_1.8 currents were enhanced (Meents et al., 2019; Renganathan et al., 2001).

The well-established interplay between CaSR and proinflammatory cytokines (Iamartino & Brandi, 2022) and the significance of the inflammatory process in neuropathic pain (Ellis & Bennett, 2013; Li et al., 2023) suggests CaSR as a potential candidate for neuropathic pain therapy. Our initial findings about the CaSR-Kv7.2/7.3 channel crosslink posited that in neuropathic pain where the activity of Kv7.2/7.3 channels was suppressed (Ling et al., 2017; Rose et al., 2011), a calcilytic might alleviate such circumstance by withdrawing CaSR-mediated inhibition of Kv7.2/7.3 channels. In our experiments, the tendency of the algogenic cocktail in inducing a complete train of iAPs was not observed when NPS-2143 also was applied, suggesting that NPS-2143 stabilised neuronal excitability and rescued the neurons from the algogenic cocktail-induced enhancement of iAP firing. This finding further supports our hypothesis that calcilytics may reduce excitability via the CaSR-Kv7.2/7.3 channel crosslink as Kv7.2/7.3 channel suppression was shown to enhance iAP firing (Peng et al., 2017).

Spike analysis of the first iAP revealed that NPS-2143 enhanced afterhyperpolarisation in the present study. Afterhyperpolarisation is mediated by Kv7.2/7.3 channels and small/intermediate-conductance Ca^2+^-activated K^+^ channels (Gu et al., 2005; Mateos-Aparicio et al., 2014; Storm, 1989; Tzingounis & Nicoll, 2008). However, Vassilev et al. (1997) demonstrated that CaSR activation increased the open-state probability of Ca^2+^-activated K^+^ channels. Therefore, NPS-2143 likely enhanced afterhyperpolarisation via Kv7.2/7.3 channels, rather than Ca^2+^-activated K^+^ channels, further supporting our theory of the CaSR-Kv7.2/7.3 channel crosslink.

Although NPS-2143 inhibited the algogenic cocktail in driving the hiPSC-derived nociceptive-like neurons to develop a complete iAP train characteristic, it appeared to synergise with the algogenic cocktail in increasing the iAP spike frequency at lower current injection of the neurons that presumably already expressed a train of iAPs. Blocking of Ca^2+^-activated K^+^ channels enhanced iAP firing in rodent DRG neurons (Pagadala et al., 2013; Tsantoulas & McMahon, 2014; Zhang et al., 2003). Based on existing evidence that CaSR activation up-regulates Ca^2+^-activated K^+^ channel function (Vassilev et al., 1997), one might speculate that NPS-2143 would inhibit Ca^2+^-activated K^+^ channels and augment iAP firing. A previous study demonstrated that most human DRG neurons produced a single iAP (Davidson et al., 2014). Therefore, when it comes to modulation of collective excitability, the effect of NPS-2143 in preventing algogenic cocktail-induced transformation of the neurons with a single iAP towards multiple iAPs may be greater than its effect in enhancing iAP firing of the cells already at the high-end of the excitability spectrum. The effect of the calcilytic on neuronal excitability, thus, is likely to be multimodal with several knowledge gaps worth pursuing in the future.

Considering the calcilytic's potential in regulating Kv7 channels and neuronal excitability, a calcilytic might help address shortcomings of direct Kv7 channel modulators. Unlike Kv7 channel openers such as flupirtine, calcilytics appeared to be safer and more tolerable (Fitzpatrick et al., 2012; Halse et al., 2014) and could be repurposed for neuropathic pain management. A combined use of the calcilytic and Kv7 channel opener is worth investigating and might reduce the required dosage of the Kv7 channel opener, thus limiting its adverse effects.

### 4.3 Limitations

First, the effect of [Ca^2+^]_o_ could be due to its off-target actions. This limitation was circumvented by using NPS-R568 to supplement our findings, and the same effect was observed. Secondly, ELISAs only gave information about overall changes within the cell but no information regarding localisation of the effects. This warrants future investigations into the effect of CaSR activation on spatially distinct cAMP levels and PKA activity. The hiPSC-derived nociceptive neuron-like model provides a promising platform to explore novel therapeutics for pain but is limited by how well the model can replicate in vivo human nociception. Our model still lacked the full complexity of in vivo models, such as the interaction with glia, which is crucial to neuropathic pain development (Zhang et al., 2017) and the complete assemblage of algogenic mediators released in vivo (Ellis & Bennett, 2013).

### 4.4 Conclusions

This study demonstrates the CaSR-Kv7.2/7.3 channel crosslink mechanism and the calcilytic's potential for controlling hyperexcitability. Our findings spearhead CaSR as a potential therapeutic target for Kv7 channel-associated hyperexcitability disorders such as neuropathic pain, which is worth further investigation.

## Supporting Information

Additional supporting information can be found online in the Supporting Information section at the end of this article.

## Data Availability

Data needed to evaluate the conclusions in the paper are present in the paper. Data generated for this study are available from the corresponding author upon reasonable request.

## What is already known?

- Neuronal Kv7 channels regulate excitability, and their dysfunction is associated with neuropathic pain.
- CaSR can modulate the activity of neuronal ion channels and thus excitability.

## What does this study add?

- Activation of CaSR inhibits Kv7 channels via G_i/o_ protein signalling.
- Calcilytic drugs can rescue hiPSC-derived nociceptive-like neurons from hyperexcitability induced by an algogenic cocktail.

## What is the clinical significance?

- CaSR may be a therapeutic target for Kv7 channel-associated hyperexcitability disorders, such as neuropathic pain.

## Abbreviations

### Abbreviations

[Ca2+]i: intracellular Ca^2+^ concentration
[Ca2+]o: extracellular Ca^2+^ concentration
AC: adenylate cyclase
ANOVA: analysis of variance
AP: action potential
ATP: adenosine triphosphate
BAPTA-AM: 1,2-bis(2-aminophenoxy)ethane-N,N,N',N'-tetraacetic acid tetrakis acetoxymethyl ester
cAMP: 3′,5′-cyclic adenosine monophosphate
CaSR: Ca^2+^-sensing receptor
Cav: voltage-gated calcium
CHO-Kv7.2/7.3: Chinese hamster ovary cells stably expressing human Kv7.2/7.3 channels
DAPI: 4′,6-diamidino-2-phenylindole
DMEM: Dulbecco’s modified Eagle medium
DRG: dorsal root ganglia
EC50: half maximal depolarisation
EDTA: ethylenediamine tetra acetic acid
ELISA: enzyme-linked immunosorbent assay
GPCR: G protein-coupled receptor
H: Hill slope
HEK-CaSR: human embryonic kidney 293 cells stably expressing the CaSR
hiPSC: human induced pluripotent stem cell
iAP: induced action potential
Im: M current
Kv: voltage-gated potassium
MAP: microtubule-associated protein
NAM: negative allosteric modulator
Nav: voltage-gated sodium
PAM: positive allosteric modulator
PBS: phosphate buffer solution
PKA: protein kinase A
PLA: proximity ligation assay
PLC: phospholipase C
PTX: pertussis toxin
RMP: resting membrane potential
sAP: spontaneous action potential
SEM: standard error of the mean
TNF: tumour necrosis factor
TRPV: transient receptor potential vanilloid
